# Nintedanib and ischemic colitis: Signal assessment with the integrated use of two types of real‐world evidence, spontaneous reports of suspected adverse drug reactions, and observational data from large health‐care databases

**DOI:** 10.1002/pds.5022

**Published:** 2020-05-12

**Authors:** Rebecca E. Chandler

**Affiliations:** ^1^ Uppsala Monitoring Centre Uppsala Sweden

**Keywords:** pharmacoepidemiology, pharmacovigilance, real‐world evidence, signal detection

## Abstract

**Purpose:**

Statistical screening of Vigibase, the global database of individual case safety reports, highlighted an association between the MedDRA Preferred Term (PT) “colitis” and nintedanib. Nintedanib is a protein kinase inhibitor authorized in accelerated regulatory procedures for the treatment of idiopathic pulmonary fibrosis (IPF). The aim of this report is to describe the integration of two types of real‐world evidence, spontaneous reports of adverse drug reactions (ADR), and observational health data (OHD) in the assessment of a post‐authorization safety signal of ischemic colitis.

**Methods:**

Assessment of the statistical signal of “nintedanib – colitis” was undertaken using data from VigiBase, OHD from the Observational Heath Data Sciences and Informatics (OHDSI) collaborative, published literature, and openly available regulatory documents. Evidence synthesis was performed to support Bradford Hill criteria in causality assessment.

**Results:**

Evidence for strength of association, specificity, consistency, and analogy was found upon review of the case series. OHD was used to calculate incidence rates of colitis in new users of nintedanib across multiple populations, supportive of consistency, and further evidence for strength of association. Literature review identified support for biological plausibility and analogy. Signal assessment was supplemented with characterization of real‐world users and exploration of potential risk factors using OHD.

**Conclusions:**

An integrated approach using two forms of real‐world data, spontaneous reports of ADRs and data from observational databases allowed a comprehensive and efficient signal assessment of nintedanib and colitis. Further exploration of the complementary use of real‐time OHD in signal assessment could inform more efficient approaches to current signal management practices.

1

**KEY POINTS**

- A causal relationship between ischemic colitis and nintedanib is hypothesized
- Complementary use of two types of real‐world evidence, spontaneous reports of ADR, and claims data from observational databases can be used in signal assessment
- “Real‐time evidence synthesis” has the potential to increase the efficiency in the system to act on post‐marketing safety signals.

## 2 INTRODUCTION

Nintedanib is a small molecule receptor tyrosine kinase inhibitor (TKI) blocking vascular endothelial growth factor receptors (VEGFR 1‐3), fibroblast growth factor receptors (FGFR 1‐3), and platelet‐derived growth factor receptors (PDGFR) α and β kinase activity. It received marketing approval from the European Medicines Agency (EMA) and the United States Food and Drug Administration (USFDA) in 2014 for the treatment of IPF, a rare disease characterized by an excess of fibroblasts, leading to progressive fibrosis of the lung. Marketing approval was granted by the EMA after an accelerated assessment and the USFDA after a priority review; less than 1000 patients received nintedanib in pivotal trials that provided the basis for licensure. 1, 2

Ischemic colitis is a condition caused by insufficient blood flow to the large intestine, which results in mucosal ulceration, inflammation, and hemorrhage. Causes are physiological (hypotension, secondary to embolus/thrombosis) or iatrogenic (secondary to medicines, surgery). Ischemic colitis manifests with diarrhea, colicky abdominal pain, and rectal bleeding. Patients with ischemic colitis should be recognized quickly, as colonoscopic evaluation is recommended within 48 hours of symptom onset. Colonoscopy can distinguish those cases that may be treated with conservative management from those that require emergency resection. The complications arising from ischemic colitis include obstruction, necrosis, and perforation.^3^

The aim of this report is to describe the assessment of a signal of colitis and nintedanib with the complementary use of spontaneous reports of ADRs and OHD.

## 3 METHODS

Statistical screening in VigiBase using the VigiRank algorithm^4^ was performed in December 2019 and highlighted the drug‐adverse event (AE) combination of “nintedanib – colitis” for review. The drug‐AE pair was assessed for the potential for a causal relationship using the Bradford Hill criteria.^5^ Additional analyses within VigiBase and several observational databases (insurance claims databases from the US) available via the Observational Heath Data Sciences and Informatics (OHDSI) collaborative,^6^ as well as reviews of the licensure application and published medical literature, were performed to explore the potential for a causal relationship between nintedanib and colitis.

## 4 RESULTS

Clinical review of the 25 individual case safety reports (ICSRs) for the drug‐AE pair revealed different types of colitis, such as ischemic or inflammatory, or was further unspecified. Given that nintedanib acts upon the VEGF receptor, the association between nintedanib and the more specific clinical concept of “ischemic colitis” was explored.

One case from the original “colitis” case series had information sufficient to be considered as “ischemic colitis” (colonoscopy results were provided). Subsequent exploration within VigiBase used the MedDRA SMQ, ischemic colitis (narrow); nine additional cases were identified. The final case series of ischemic colitis included a total of 10 cases coded to different MedDRA PT: five cases of “colitis ischemic,” two of “intestinal ischemia,” one reporting both “colitis ischemic” and “intestinal ischemia,” one of “intestinal infarction,” and one of “colitis.”

Within these 10 reports, seven reports described males and three described females (Table 1). Ages ranged between 53 and 78; age was not included in one report. Reports originated from five countries (one country in the Americas, three countries in Europe, and one country in Asia). Time to onset was from 3 days to 5 months. In seven cases, the indication for nintedanib was IPF; in the three other cases, non‐small cell lung cancer, bronchial carcinoma, and glioblastoma. Diagnostic results were reported in three cases (6, 8, and 10). Fatal outcomes were reported in four cases. Limited additional information was available in most cases given that narratives had not been shared into Vigibase. Potential confounding/risk factors for ischemic colitis were advanced age, concomitant use of corticosteroids in two cases (1 and 5) and medical history of hyperlipidemia in two cases (2 and 5).

**TABLE 1:** Individual case safety reports included in the relevant case series identified from Vigibase

Case	Age/sex	Drugs	Indication	ADR	Dosage	TTO	Outcome	Notes
1	78y/M	Nintedanib (S) Prednisolone (S) Acetylcysteine (C) Dimemorfan (C) Drug not accepted in WHODrug (C)	Idiopathic pulmonary fibrosis	Ischemic enterocolitis	300 mg qd × 2 months, 200 mg qd × + months	15 days	Recovered	55 kg, 159 cm Report from study
2	74y/M	Nintedanib (S) Vonoprazan (C) Irbesartan; Trichlormethiazide (C) Atorvastatin (C) Acetylsalicylic acid (C)	Idiopathic pulmonary fibrosis	Colitis ischemic Pneumonia bacterial	300 mg qd × 11 months 200 mg × qd × 2 months	3 days	Recovered	62.9 kg, 165 cm Report from study
3	65y/F	Nintedanib (S) Alfacalcidol (C) Levothyroxine (C) Ascorbic acid; pantothenic acid (C) Minodronic acid (C) Drug not accepted in WHODrug (C)	Idiopathic pulmonary fibrosis	Colitis ischemic	300 mg qd × 10 months	4 months	Not recovered	50.5 kg, 161 cm Report from study
4	77y/M	Nintedanib (S) Insulin (C)	Idiopathic pulmonary fibrosis	Colitis ischemic Abnormal loss of weight Septic shock Decreased appetite Vomiting Nausea Diarrhea	150 mg 2 per day		Died	
5	78y/F	Nintedanib (S) Tacrolimus (C) Prednisolone (C) Trimethoprim/sulfamethaxazole (C) Warfarin (C) Alfacalcidiol (C) Rabeprazole (C) Loperamide (C) Drug not accepted in WHODrug (C) Drug not accepted in WHODrug (C) Drug not accepted in WHODrug (C) Drug not accepted in WHODrug (C)	Idiopathic pulmonary fibrosis	Ischemic enterocolitis	200 mg qd × 5 months	5 months	Died secondary to ischemic entercolitis and lower gastrointestinal perforation	36 kg/153 cm Report from study. History of hyperlipidemia, osteoporosis, gastroesophageal reflux disease, venous thrombosis, constipation, insomnia, chronic gastritis, and breast cancer
6	53y/M	Nintedanib (S)	Glioblastoma	Mesenteric ischemia Colitis ischemic Obstipation Stomach pain Thromboembolic event Ischemia peripheral Wound healing delayed Wound infection	400 mg per day	27 days	Died	185 cm Report from study. Patient presented with abdominal pain 27 days after initiation on nintedanib. Admitted to hospital, diagnosed with ischemic colitis (grade 2), and treated with mesalazin. Five days later, the patient experienced obstipation. Ten days later, the patient had stomach pain (grade 3), acute mesenteric ischemia (grade 4), thromboembolic event (grade 4), and peripheral ischemia (grade 3). Had thromobectomy of arteria mesenterica superior, explorative laparotomy, and small intestinal segment resection. Hospitalization complicated by wound healing disorder, which led to cardiovascular failure, sepsis, and death.
7	‐/M	Nintedanib (S) Omeprazole (S) Indacaterol (C) Olmesartan (C) Salbutamol (C) Lercanidipine (C)	Fibrosis lung	Bowel ischemia Vein thrombosis mesenteric	2 DF per day		Recovering	
8	56y/F	Nintedanib (S) Docetaxel (S) Zoledronic acid (C) Levothyroxine (C)	Bronchial carcinoma	Intestinal ischemia Bowel ischemia Gastrointestinal necrosis Necrosis bowel	400 mg per day	20 days	Unknown	Surgery preparation of colon descendens, sigmoideum, and upper rectum with intense and distinct ischemic necrosis of gut wall, inclusion of max. 2.3 cm polyp, without evidence of vital mucosa. Beside a 0.9 cm in sano resected tubular adenoma with slight intraepithelial neoplasia (low‐grade adenoma following WHO‐classification). Transmural hemorrhagic necrosis (reaching out up to the resection area) of gut wall without inflammation, as appropriate for ischemia.
9	74y/M	Nintedanib (S) Lansoprazole (C)	Idiopathic pulmonary fibrosis	Intestinal infarction			Died	69 kg
10	57 y/M	Nintedanib (S) Ipratropium (C) Acetylsalicyclic acid (C) Glyceryl trinitrate (C) Fluvastatin (C) Torasemide (C) Lormetazepam (C) Metamizole (C) Omeprazole (C)	Non‐small cell lung cancer	Colitis Diarrhea Nausea Mucositis Anorexia Respiratory failure		9 days		Serious. Clinical trial patient. Died, cause of death “respiratory failure” Medical history: Idiopathic cardiomyopathy Bronchial disorder Insomnia Pain Smoker Alcohol abuse Patient experienced diarrhea and vomiting which required hospitalization 10 days after initiation of drug. CT scan of abdomen on day of admission with probably stenosis lesion of the sigmoid colon and proximal colonic dilation and loops of small bowel. Colonoscopy 11 days after discontinuation of drug revealed ischemic colitis. Repeat CT 16 days after discontinuation revealed no evidence of obstruction. Investigator considered that there is a causal relationship between the drug and GI symptoms and signs.

(C), drug concomitantly administered with suspected drug. (S), drug suspected to be associated with reported adverse drug reaction (ADR).

Observational data were used to explore “real‐world use” of nintedanib after marketing approval. Use of nintedanib started in 2014 with most users in the 70‐ to 80‐year age range and in more males than females. New users of nintedanib had no prior history of colitis and did not significantly differ from users of the alternative therapy for IPF, pirfenidone.

Further signal assessment included synthesis of data from ICSRs and the ODH as well as published literature in the consideration of the Bradford Hill criteria. The ***biologic plausibility*** is supported by the inhibitory action of nintedanib on the VEGF receptor. VEGF is a protein that mediates multiple functions within the vascular system, including endothelial cell proliferation as well as vascular permeability and vasodilation.^7^ A mouse model has shown that inhibition of VEGF signaling resulted in regression of capillaries of intestinal villi,^8^ and VEGF inhibition could contribute to perforation by inducing regression of normal blood vessels in the gastrointestinal tract^9^ (presumptively via an intermediate step of ischemia). ***Specificity*** was explored in the spontaneous database using the MedDRA SMQ “ischemic colitis”; evidence of disproportionality was found for multiple PTs within this SMQ, including “colitis ischemic” (IC025 0.32), “large intestine perforation” (IC025 1.67), and “large intestinal hemorrhage” (IC025 0.39). Evidence for ***strength of association*** is found in the disproportionality in Vigibase and measures of incidence from observational data (eg, 56.74 cases per 1000 person‐years from one insurance system). ***Consistency*** is found in both ICSRs (reports from numerous countries) and OHD (increased incidence observed in multiple insurance systems). A consideration of ***analogy*** is feasible given the availability of other antiangiogenic agents which antagonize VEGF (aflibercept and bevacizumab) or inhibit the VEGF receptor (regorafenib and sorafenib). Literature review reveals case reports of ischemic colitis with aflibercept^10^ and bevacuzimab,^11^ both administered intravitreally. Data mining within Vigibase reveals disproportional reporting of MedDRA PT within the “ischemic colitis” SMQ for a number of other analogous agents: (bevacizumab: intestinal ischemia 2.23; colitis ischemic 2.18; aflibercept: colitis ischemic 0.66; midostaurin: intestinal ischemia 0.42; ranibizumab: intestinal ischemia 0.38; sunitinib: intestinal ischemia 0.23; and sorafenib: colitis ischemic 0.18).

Additionally, OHD could allow for a preliminary estimation of the incidence risk ratio from a self‐controlled “pre‐exposure” comparative cohort.

## 5 DISCUSSION

Complementary use of two types of real‐world evidence, ICSRs and ODH, supports the hypothesis of a causal relationship between nintedanib and ischemic colitis. Integration of evidence from the case series and explorations of observational data in “real‐time” allowed for a comprehensive and efficient review of the points to consider in signal assessment, such as a characterization of real‐world users, exploration of potential confounders, and evidence in support Bradford Hill causality criteria. Communication of this signal is considered warranted as early identification of ischemia may prevent progression to the serious, life‐threatening event of gastrointestinal perforation. Given that the most commonly occurring gastrointestinal adverse reaction for nintedanib is diarrhea, which can be a symptom of ischemic colitis, it could be important to inform health‐care providers to rule out ischemia prior to the recommended symptomatic treatment of diarrhea with loperamide.

An important limitation in this pilot study was the differences in the amounts of data and medical terminology between the different data sources. For example, further specification of “colitis” was not possible in the OHD due to the relatively small number of patients taking nintedanib. Furthermore, clinical reasoning is required to translate signals identified in MedDRA terminology into explorations of databases using ICD coding.

Several publications have explored different approaches to the use of observational data in signal detection. A large European initiative, the EU‐ADR project, has explored the use of real‐world healthcare data in signal detection; the findings revealed that potential for signal detection within real‐world data was found to be dependent on frequency of the event and utilization of drugs in the general population.^12^ Other proposals have included a signal detection strategy that requires signaling from both an adverse event reporting system and electronic health records^13^ and the use of electronic medical records for placing signals, which have been highlighted by individual case safety reports, within clinical context. ^14^

As noted in a piece by Trifuro et al, “To harness the potential of big data in pharmacovigilance, it is important to use complementary methods and data sources that play on their respective strengths.”^15^ This signal assessment is intended as a proof‐of‐concept example of how an analysis of individual cases and observational data can be synthesized into evidence to support a causal hypothesis. Further exploration of the complementary use of observational data in signal assessment should be performed to inform more efficient approaches to current signal management practices.

## CONFLICTS OF INTEREST

The opinions expressed in this piece are not necessarily those of the national pharmacovigilance centers of the WHO Program for International Drug Monitoring or of the WHO. The author has no conflicts of interest that are directly relevant to the content of this piece.
